# Nonlinear Elasto-Mammography for Characterization of Breast Tissue Properties

**DOI:** 10.1155/2011/540820

**Published:** 2011-12-22

**Authors:** Z. G. Wang, Y. Liu, G. Wang, L. Z. Sun

**Affiliations:** ^1^Department of Civil and Environmental Engineering, The University of Iowa, Iowa City, IA 52242, USA; ^2^Prudential Insurance Company, Newark, NJ 07102, USA; ^3^Cooper Tire and Rubber Company, Findlay, OH 45840, USA; ^4^School of Biomedical Engineering and Sciences, Virginia Tech, Blacksburg, VA 24061, USA; ^5^Department of Civil and Environmental Engineering, University of California, Irvine, CA 92697-2175, USA

## Abstract

Quantification of the mechanical behavior of normal and cancerous tissues has important implication in the diagnosis of breast tumor. The present work extends the authors' nonlinear elastography framework to incorporate the conventional X-ray mammography, where the projection of displacement information is acquired instead of full three-dimensional (3D) vector. The elastic parameters of normal and cancerous breast tissues are identified by minimizing the difference between the measurement and the corresponding computational prediction. An adjoint method is derived to calculate the gradient of the objective function. Simulations are conducted on a 3D breast phantom consisting of the fatty tissue, glandular tissue, and cancerous tumor, whose mechanical responses are hyperelastic in nature. The material parameters are identified with consideration of measurement error. The results demonstrate that the projective displacements acquired in X-ray mammography provide sufficient constitutive information of the tumor and prove the usability and robustness of the proposed method and algorithm.

## 1. Introduction

Breast cancer is a major threat to public health in the world. In USA and Europe, approximately 10% of women develop breast cancer during the course of their lives. While the specific causes of breast cancer are unknown, early detection and characterization of breast tumors is the key to successful treatment. Currently, X-ray mammography, a low-dose X-ray imaging modality, is the primary diagnosis method in clinics [1]. While being more efficient in detecting malignancies as age increases or the breast becomes fatty, mammography fails to identify small cancers in dense breasts. Furthermore, mammography may not be specific in terms of tumor benignity and malignancy. About 80% of suspicious masses referred by mammography for surgical breast biopsy are in fact not malignant [2–4]. These false-positive mammograms may induce patients' anxiety, distress, and intrusive thoughts.

A number of techniques have been attempted to address these problems associated with mammography. From the viewpoint of mechanics, the tissue stiffness is an important index for diagnosis of breast cancers, as tumors are stiffer than the surrounding breast tissues and malignant tumors are much stiffer than benign ones [5–7]. In other words, in vivo identification of the mechanical parameters of normal and abnormal tissues should improve the accuracy of cancer diagnosis. Correspondingly, elastography has been proposed as a method to image the tissues' elasticity in a quantitative manner. The general basis of elastography is to induce motion within tissue by mechanical stimulation. Conventional medical imaging modalities are then used to measure the spatial deformation, from which the mechanical properties can be extracted. Based on the imaging modalities used, elastography has two major classes: ultrasound elastography (USE) and magnetic resonance elastography (MRE). USE, developed in the 1990s, is the first modulus-imaging modality. It computes the lap between the pre- and postcompression radio frequency ultrasound signals to estimate the tissue's axial displacement and strain under quasistatic loading [8, 9]. While providing new information for detecting pathological tumors, USE suffers from limited stiffness range as imposed by the minimum resolvable wavelength. The computed image in USE is also restricted by the angular resolution of the transducer and its ability to separate signals from artifacts and noise [9]. Magnetic resonance elastography (MRE) is a second-generation elastography modality that provides higher resolution images and is capable of producing sufficient 3D spatial and contrast resolution [10, 11]. MRE is, however, significantly more costly as a result of the MR imaging procedure and hence is not generally applicable for all patients. From the viewpoint of solid mechanics, the current USE and MRE are insufficient, because both are based on infinitesimal-strain linear elasticity and only very few are capable of considering anisotropic tissue properties. In other words, the large deformation, nonlinear, and anisotropic behaviors of breast tissues (fat and glandular tissues) and tumor have not yet been taken into consideration by USE or MRE. Therefore, the outcomes of USE and MRE may not be sufficiently accurate for the diagnostic purpose.

Motivated by the significance of early detection of breast tumors and the current limitations of mammography and elastography modalities, we have developed a nonlinear elasto-mammography method that takes into consideration of the finite-strain nonlinear properties of breast tissues, in combination with mammography visualization. The development has experienced two stages.

First, a *linear elasto-mammography* framework was developed to generate the elastograms of breast tissues, by combining the conventional low-dose X-ray mammography with linear elastography framework [12]. Instead of applying ultrasound or magnetic resonance as in the previous elastography research, elasto-mammography uses displacement information extracted from mammography projections before and after breast compression. Incorporating the displacement measurement, an elastography reconstruction algorithm was specifically developed to estimate the elastic moduli of heterogeneous breast tissues. Case studies with numerical breast phantoms showed that the displacement measurement obtained from mammography is sufficient to identify the material parameters of breast tissues and tumors within the framework of linear elasticity.

Then, a *nonlinear elastography* method was proposed [13]. As discussed above, the current elastography (USE or MRE) reconstruction framework is based on the assumption of linear elasticity theory. The mechanics of biological soft tissues, however, require nonlinear continuum mechanics description [14, 15]. While tissue models based on linear elasticity have been broadly used, they are reliable only when the tissue strain is less than 5% [16], which is much lower than the deformation of soft tissues. Thus, consideration of nonlinearity is essential for elastography in clinical applications. Our development of nonlinear elastography method, for the first time, enables identification of the mechanical properties of soft breast tissues and tumor. To improve the computational efficiency and enhance the stability, a nonlinear adjoint method was introduced. The phantom study demonstrated that the complex nonlinear mechanics of soft breast tissues and tumors can be quantified from 3D displacement and force measured on the surface of the breast.

The objective of the present study is to develop a *nonlinear elasto-mammography* framework that combines the simplicity of projective X-ray mammography measurement with the accuracy of nonlinear elastography. In Section 2, we present the mathematical derivation, where an adjoint gradient method is modified to consider the projective displacement measurements. Finite-element- (FE-) based numerical simulations are conducted in Section 3 to reconstruct the material parameters of a 3D heterogeneous breast phantom from mammography displacement. Two types of mammography compressive loadings are applied, and the displacements at key points on the tissue interfaces are extracted from mammography projections before and after deformation. In Section 4, the results are presented and the effect of experiential error is investigated.

## 2. Methods

### 2.1. Finite-Strain Deformation Equations

Let *Ω*^0^ be a biological object subjected to body force **b** and surface force **t** on boundary Γ_**t**_^0^. Here, we consider general problems that the body force **b** and surface force **t** are deformation dependent. Following the standard finite-element method, the displacement **u** is discretized as nodal displacement vector {*u*} = {*u*_1_,  *u*_2_}^*T*^, where *u*_2_ corresponds to $\overset{®}{u}$ prescribed on Γ_**u**_^0^ and *u*_1_ is to be solved from nonlinear equations; that is, on surface Γ_**u**_^0^ (Γ_**u**_^0^ ∪ Γ_**t**_^0^ = ∂*Ω*^0^), as described in [13], the FE description of the finite-strain equilibrium equation is

(1) $$\left\{\begin{array}{c} f_{1}^{\text{in}}\left(u_{1},u_{2};p\right)\\ f_{2}^{\text{in}}\left(u_{1},u_{2};p\right) \end{array}\right\}-\left\{\begin{array}{c} f_{1}^{\text{out}}\left(u_{1},u_{2}\right)\\ f_{2}^{\text{out}} \end{array}\right\}=\left\{\begin{array}{c} 0\\ 0 \end{array}\right\}.$$

The internal nodal force *f*^in^ corresponds to the stress of the tissue; that is, it changes with *u*_1_ and material parameters **p** but not *u*_2_ as it is prescribed. The external nodal force *f*_1_^out^ is due to the prescribed surface force **t** and body force **b** in biological object *Ω*^0^. It changes with the displacement in large deformation. The nodal force *f*_2_^out^ is the unknown constraint force on Γ_**u**_^0^.

A classic quasi-Newton method [17] is employed to solve (1) for *u*_1_. Let *u*_1_^(*n*)^ be the trial solution of the unknown *u*_1_ at the *n*th iterative step. An improved solution *u*_1_^(*n*+1)^ = *u*_1_^(*n*)^ + *δu*_1_ can be obtained at the next step, in which *δu*_1_ is the solution of linear equations:

(2) $$\begin{array}{c} K_{11}^{\text{eff}}\left(u_{1}^{\left(n\right)}\right)\delta u_{1}=f_{1}^{\text{in}}\left(u_{1}^{\left(n\right)},u_{2};p\right)-f_{1}^{\text{out}}\left(u_{1}^{\left(n\right)},u_{2}\right) \end{array}$$

with

(3) $$\begin{array}{c} K_{11}^{\text{eff}}=\left(K_{11}^{\text{in}}-K_{11}^{\text{out}}\right),\;\;K_{11}^{\text{in}}=\frac{\partial f_{1}^{\text{in}}}{\partial u_{1}},\;\;K_{11}^{\text{out}}=\frac{\partial f_{1}^{\text{out}}}{\partial u_{1}}, \end{array}$$

where the matrices are evaluated at *u*_1_^(*n*)^.

### 2.2. Nonlinear Elasto-Mammography Algorithm

We consider that the biological object *Ω*^0^ is discretized into FE mesh, and the displacement and force are discretized consistently into nodal displacement and nodal force. Experimental measurement for elasto-mammography is displacement. We catalog the measurements as the following. (i) If the force at a node is known, it will be included into *f*_1_^out^ which is considered “prescribed” in (1). The corresponding nodal displacement will be considered as unknown *u*_1_ in the FE equation (1). (ii) All the other nodal displacements will be in *u*_2_, and the corresponding unknown nodal force will be in *f*_2_^out^. For category (ii), *u*_2_ must be considered “prescribed” to fulfill the requirement of the well poseness of a solid mechanics problem.

In our previous elastography method [13], displacements are also measured at some of the nodes associated with *u*_1_ and are denoted as *U*_1_^*M*^. Given material parameters **p**, the unknown displacement *u*_1_ and constraint force *f*_2_^out^ (which depends on **p**) will be solved from the FE equation (1). The elastography method thus seeks **p** so that the overall difference between measured *U*_1_^*M*^ and computed *u*_1_ is minimum; that is, to minimize objective function:

(4) $$\begin{array}{c} \Phi \left(p\right)={\left(u_{1}^{}-U_{1}^{M}\right)}^{T}\Lambda \left(u_{1}^{}-U_{1}^{M}\right), \end{array}$$

where diagonal weight matrix Λ = diag⁡(*a*_1_, *a*_2_,…, *a*_*j*_,…), with component *a*_*j*_ = 1 when the *j*th component of *U*_1_^*M*^ is experimentally measured, or *a*_*j*_ = 0 otherwise.

In mammography, however, the measurement of displacement is limited by the projection; that is, only the two components perpendicular to the projection direction are obtainable. Correspondingly, the computed displacement *u*_1_ should be *projected* in the same direction as in mammography and then compared with the mammography measurement *U*_1_^*M*^. As derived in Appendix  A, the projection can be represented by a linear translation of *u*_1_, as **R***u*_1_, where **R** is a global projection matrix. The objective function for nonlinear elasto-mammography is then

(5) $$\Phi \left(p\right)={\left(Ru_{1}-U_{1}^{M}\right)}^{T}\Lambda \left(Ru_{1}-U_{1}^{M}\right).$$

### 2.3. Nonlinear Adjoint Method

Efficient and robust optimization-based elastography reconstruction schemes request user-supplied gradient ∂Φ/∂**p**. Direct calculation of the gradients ∂Φ/∂**p** involved in the minimization-based parametric identification is difficult, because *u*_1_ is an implicated function of **p**. Recently, an adjoint method was introduced to compute the gradient analytically [18–21]. The corresponding nonlinear finite element formulas are shown in Appendix  B. Briefly, given a trial **p**, *u*_1_ will be solved from FE equations (2) and (3), the objective function will calculated by (5), and the material parameters **p** will be updated by large-scale limited memory BFGS (L-BFGS) method with user supplied gradients readily obtained as:

(6) $$\frac{\partial \Phi }{\partial p}={\left\{\begin{array}{c} w_{1}\\ w_{2} \end{array}\right\}}^{T}\left\{\begin{array}{c} \frac{\partial f_{1}^{\text{in}}}{\partial p}\\ \frac{\partial f_{2}^{\text{in}}}{\partial p} \end{array}\right\},$$

where the virtual adjoint displacements *w*_1_ and *w*_2_ are solved from linear equations:

(7) $$\begin{array}{c} K_{11}^{\text{eff}}w_{1}=-2R^{T}\Lambda \left(Ru_{1}-U_{1}^{M}\right),\\ w_{2}=0, \end{array}$$

with the tangent stiffness matrix *K*_11_^eff^ defined in (3). The most significant features of the adjoint method are the analytical formulation, high accuracy, and computational efficiency [22]. Since *K*_11_^eff^ and its LU factorization have been calculated when solving the FE equation (2), the additional computational expense for *w*_1_ in (7) is minimal. Furthermore, it only needs to solve one linear equation (7) regardless of the number of unknown parameters in **p**.

**Figure 1:**
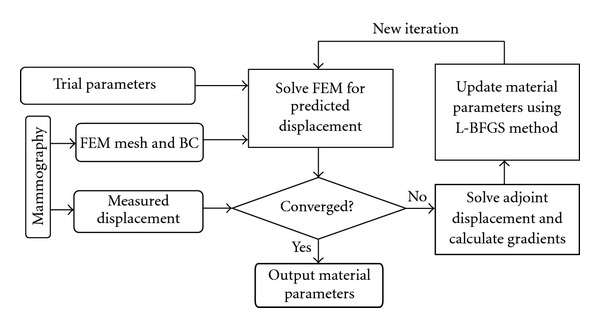
Overall flowchart for nonlinear reconstruction of material parameters of breast tissues.

The reconstruction procedure is illustrated in Figure 1. We first establish a numerical FE model of the breast tissue on which external loadings are applied. In order to measure displacement, we compare the mammography projections before and after the deformation. Then, initial guess of the distribution for material parameters (*λ*, *μ*, *γ*) is given. Given the external loadings and material parameters, the displacement filed *u*_1_ is solved from (1) and is projected to **R***u*_1_ according to the mammography direction. The difference between prediction **R***u*_1_ and measurement *U*_1_^*M*^ are evaluated by the objective function (5). The adjoint field *w* is calculated by (7), and gradients ∂Φ/∂**p** are obtained by (6). The material parameters could be updated by limited-memory BFGS (L-BFGS) optimization subroutine [23]. The iteration continues until a minimization is reached.

## 3. Numerical Simulations

### 3.1. Breast Phantom and Forward Problem

**Figure 2:**
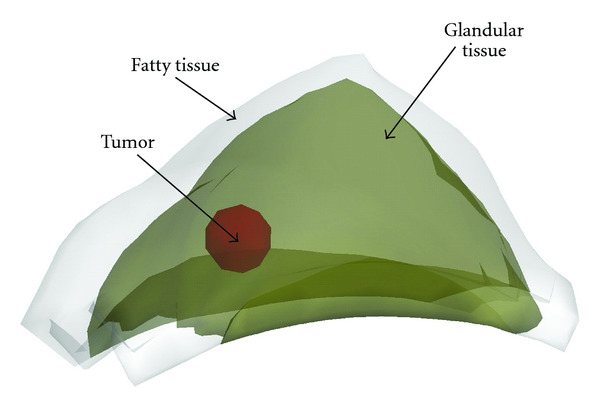
Mammography projections for 3D heterogeneous breast phantom after deformation. Fatty tissue, glandular tissue, and a tumor are shown.

We establish a 3D typical breast FE phantom, shown in Figure 2, consisting of the fatty and glandular tissues and a ductal carcinoma (tumor). Boundaries of these regions are described with sets of splines. The mechanical properties of these tissues are described with Fung-type isotropic hyperelastic model [14], whose strain energy function reads

(8) $$\;W\left(E\right)=\frac{\gamma }{2}\left[\exp\left(\lambda {\left(I:E\right)}^{2}+2\;\mu E:E\right)-1\right],$$

where **E** is the Green strain and {*λ*, *μ*, *γ*} are material parameters. The parameters {*λ*, *μ*, *γ*} are previous determined [13] from ex vivo experimental data of Samani and Plewes [24] as *λ*_*d*_ = 80, *μ*_*d*_ = 35, *γ*_*d*_ = 1.5 (*λ* and *μ* are dimensionless, *γ* is in kPa) of ductal carcinoma, *λ*_*f*_ = 35, *μ*_*f*_ = 12.5, *γ*_*f*_ = 0.4 of fatty tissue, and *λ*_*g*_ = 50, *μ*_*g*_ = 25, *γ*_*g*_ = 0.25 glandular tissue.

Motivated by the breast compression in X-ray mammography, we designed two loadings as detailed in [13]. In the FE model, the base of the breast phantom is fixed. Two paddles are used to apply displacement on the upper surface of the breast. The paddle close to tumor applies tilted compression, and another paddle is fixed to restrict the breast. 

### 3.2. Acquisition Projection Data

**Figure 3:**
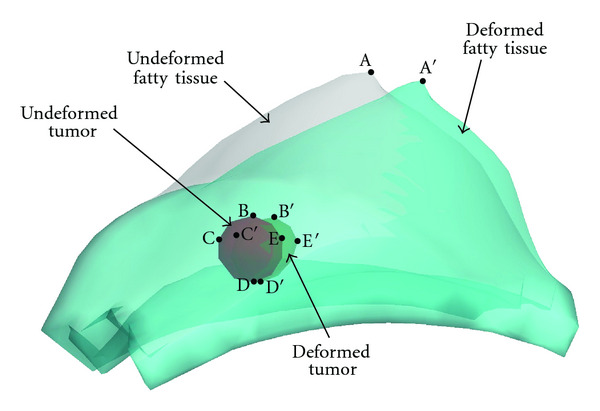
Overlapped mammography-type projections of the fatty tissue and tumor in deform and undeformed configuration. In the projections, vertexes A–E in underformed projection move to A′–E′ in deformed projection, respectively, giving the projected displacements of these points.

**Figure 4:**
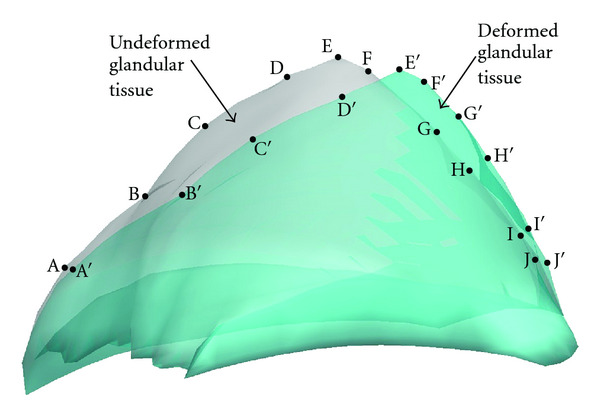
Overlapped mammography-type projections of deformed and undeformed glandular tissue. In the projections, ten nodes A–J on the surface of glandular in underformed projection move to A′–J′ in deformed projection, respectively, giving the projected displacements of these nodes.

For each loading, mammography projections for 3D heterogeneous breast phantom are taken before and after deformation (Figure 2). To mimic the displacement obtainable from mammography, we extract the displacement components in the projection plane at some discrete material points (Figure 3), denoted as *U*_1_^*M*^. We select three mammography projection directions. With each direction, one projection is made at undeformed state, and one is made at deformed configuration (Figure 2). Then, the displacement components on the projection plane are extracted from a set of landmarks in the tissues by comparison their position in undeformed and deformed projections, as shown in Figures 3 and 4. The landmarks include the top vertex on the upper breast surface (point A in Figure 3), four vertexes of the tumor surface (points B–E in Figure 3), and ten material points on the fat-glandular interface (points A–J in Figure 4). It is noted that the surfaces of tumor and glandular tissue are not smooth so that there are plenty of landmarks that can be used to track the deformation.

To explain the procedure, we use a mammography compression as example. Figure 2 shows mammography projection taken in the same direction with compression applied on the breast. The boundary of the fatty tissue, glandular tissue, and a tumor can be seen in the projection. Then, displacement components on the projection plane can be extracted by comparing the undeformed and deformed projections (Figures 3 and 4). More specifically, the undeformed and deformed projections of fatty tissue and the tumor are registered and shown together for the comparison. The top vertex of fatty tissue, point A, moves to vertex A′ after deformation. Points B–E are vertexes of the tumor in undeformed projection, and they move to vertexes B′–E′ after deformation (Figure 3). On the fat-glandular surface, we select additional ten landmarks that move from A–J to A′–J′, respectively (Figure 4). Thus, by measuring the vector from a point to its deformed position, for example, A→A′, the projective displacement components are obtained and recorded as *U*_1_^*M*^. In addition, it is assumed that there is no slip between the paddles and breast surface during mammography compression. Therefore, the displacement of the material points directly compressed by the paddles is considered known and is added to the measurement *U*_1_^*M*^.

In summary, we have obtained the following displacement measurements from mammography compression: (i) the top vertex on the upper breast surface and four vertexes of the tumor; (ii) ten nodes on the fat-glandular interface; (iii) material points directly compressed by the paddles. These displacement measurements are denoted as *U*_1_^*M*^ and will be used to identify the material parameters of the tissues.

### 3.3. Identification of Material Parameters from Displacement Measurements

Having obtained measurement *U*_1_^*M*^ from mammography compression, the inverse problem will be conducted to identify the material parameters **p** = {*λ*_*f*_, *μ*_*f*_, *γ*_*f*_, *λ*_*g*_, *μ*_*g*_, *γ*_*g*_, *λ*_*d*_, *μ*_*d*_, *γ*_*d*_} of the breast tissues and tumor, with use of an iterative optimization procedure (Figure 1). A homogeneous initial guess of *λ*_0_ = 20, *μ*_0_ = 10, *γ*_0_ = 1  (*λ* and *μ* are dimensionless, *γ* is in kPa) is used for all the materials. With a trial **p**, the displacement field *u*_1_ is solved from the FE equation (1) and is projected to **R***u*_1_ according to the mammography direction. The difference between prediction **R***u*_1_ and measurement *U*_1_^*M*^ is evaluated by the objective function Φ(**p**) (5). The gradients ∂Φ/∂**p** are computed with the proposed nonlinear adjoint method. Then, a modified trial **p** will be obtained according to the present Φ and ∂Φ/∂**p** by using L-BFGS minimization subroutine [23]. The iteration continues until a minimization is reached, which corresponds to identified material parameters.

## 4. Results and Discussion

### 4.1. Ideal Input

Table 1 shows the initial estimate and reconstructed results, together with the real values for comparison. The results in the first part are based on the ideal input. It is demonstrated that the reconstructed results are very close to the real values. The maximum error is 0.3% (*γ* for tumor) since the effect of the tumor on surface force measurement is the smallest. Reconstructions using different initial estimates have been conducted and very similar results are found, which indicates the efficiency and uniqueness of the proposed nonlinear elasto-mammography using projective measurements. In our study, all numerical experiments reached convergence and had similar convergent profiles. The iteration speed is related with initial estimations. In clinical practice, the initial estimates could be selected based on data of previous patients and experiments. The more reasonable the initial estimates are, the faster the solver got convergence.

In nonlinear elastography [13] and this study, the same nonlinear material model and properties are applied. For ideal input, both frameworks can get convergence and the reconstructed results are very close to the real values. For input with noises, both frameworks could get convergence and have the similar profiles. The parameters in fatty and glandular tissues get convergence faster than these in tumors because the fatty and glandular tissues have bigger impact on surface deformation and measurement.

**Figure 5:**
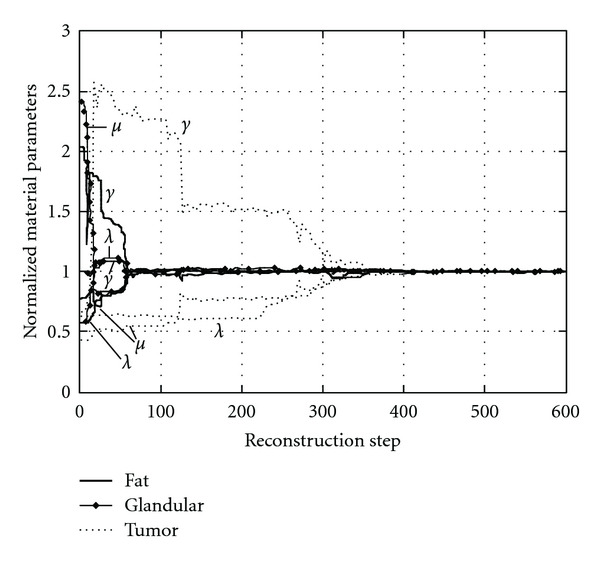
Convergent loci of elasto-mammography reconstruction for elastic parameters (*λ*, *μ*, *γ*) of fatty tissue, glandular tissue, and tumor, normalized with respect to the real values correspondingly.

Convergent loci of the elastic parameters (*λ*, *μ*, *γ*) is plotted in Figure 5. It is observed that elastic parameters of fatty tissue and glandular tissue approach the real values rapidly. After about 50 iteration steps, their relative errors are well within the range of 5%. Then, they experience some minor adjustment. In contrast, elastic parameters of the tumor converge slower. They start to fall to the real values after 300 steps. After 350 steps, all parameters are accurately identified. Reconstructions using different initial estimates have been conducted. Very similar convergent profiles are found, and equally accurate results are obtained. This indicates uniqueness of the proposed elasto-mammography for nonlinear breast tissue properties and efficiency of the reconstruction algorithm.

 The slower convergent speed of elastic parameters of the tumor is explained by the roles they play in the deformation due to the applied loadings, as discussed by Liu et al. [18]. In general, parameters with the most significant influence on the deformation are those most easy to identify. The influence of a parameter depends on size and location of the material region it belongs to, as well as characteristics of the deformation. For the present simulations, elastic parameters of fatty tissue and glandular tissue are dominant; those of tumor are much less influential, due to the small size and deep location of the tumor. So parameters of fatty tissue and glandular tissue are more accurately and easily identified than those of the tumor (Figure 5). Therefore, for successful characterization of the tumor, it is critical to apply deformation modes and acquire displacement data that are most affected by the tumor. In this elasto-mammography simulation, displacements of key points on the tumor are extracted from mammography projections, which increase the accuracy and efficiency to reconstruct the elastic parameters, especially for the tumor.

### 4.2. Multiple Sets of Measurements

Because of the nonuniqueness nature of most inverse problems, it is important to obtain sufficient measurements to reduce the likelihood of nonuniqueness. For 2D isotropic elastography, Barbone and Bamber [25] have shown that one set of displacement and force measurement, especially when measured only on the boundaries, may not provide sufficient information for reconstruction of the distribution of elastic modulus. To enhance the uniqueness of inverse problems, Barbone and Gokhale [26] proposed the feasibility of using multiple displacement fields, and Liu et al. [18] further discussed the use of multiple sets of measurements in 3D anisotropic media. In our previous nonlinear elastography study [13], measurements from four independent titled compression loadings were used to insure stable and unique material parametric reconstruction. In this work, we applied only projective measurements from two breast compression tests and found that the acquired displacement and force data are sufficient for stable parametric reconstruction, even for the small and deeply embedded tumor. This is a significant reduction, as it increases the clinical efficiency, reduces X-ray dose and operation cost, and benefits the patients.

The reduction of necessary loadings is possible because mammography projection provides displacement on the surface of the tumor, which contains direct information of the mechanics of the tumor. Our previous nonlinear elastography study [13] takes only measurement on the breast surface as input. The lack of necessary constitutive information of the tumor in the surface measurement must be compensated by increasing the number of required loadings. In case that the measurement may contain experimental errors, we must use four loadings in the elastography study, instead of two in the present elasto-mammography.

### 4.3. Iteration Steps

**Figure 6:**
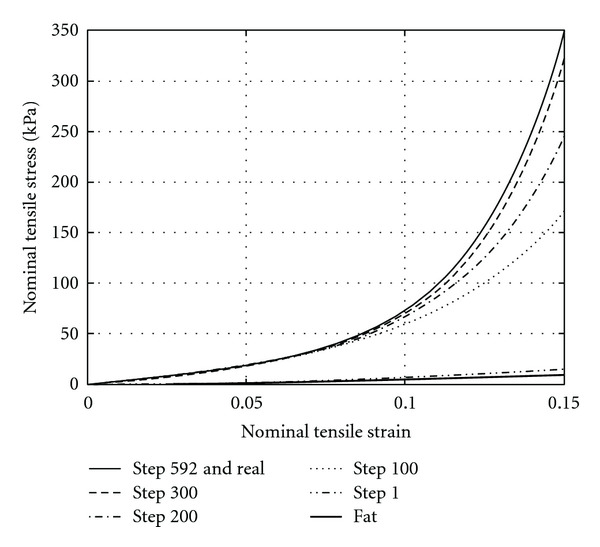
Nonlinear tensile strain-stress curves of the tumor as reconstructed at different iteration steps.

The nonlinear elasto-mammography reconstruction uses an iterative optimization procedure (Figure 1), which is controlled by user-defined criteria. This study employs more strict criteria than in our previous work [13], and it takes about 590 steps to reach the converged reconstruction results. To demonstrate the intermediate results, the uniaxial tensile strain-stress curves of the tumor predicted by the updated material parameters are plotted in Figure 6 at the 1st, 100th, 200th, 300th, and 592nd iterative steps and compared to the real one. It is observed that the reconstructed strain-stress curve approaches the real one rapidly in first 300 iterative steps. After that, the reconstruction only applies some minor adjustment.

 In clinical practice, a more tolerable criterion may be applied to control the iterative reconstruction procedure to save computational expense and time. It has been recognized that tissue stiffness plays an important role for diagnosis of breast cancers, as tumors are stiffer than that surrounding breast tissues, and malignant tumors are much stiffer than benign ones [6]. In another word, the stiffness ratio between fatty tissue and tumor, instead of real material parameters, could be used to determine the character of tumors. It is observed in Figure 6 that, starting from the 100th iterative step, the stiffness ratio of tumor to fatty tissue (the lowest curve) increases rapidly, indicating that the predicted mechanical properties of the tumor are well distinguished from the normal tissues for characterizing the tumor. Therefore, from clinical point of view, the iterative reconstruction procedure could be stopped after about 100 steps.

### 4.4. Input with Noise

**Figure 7:**
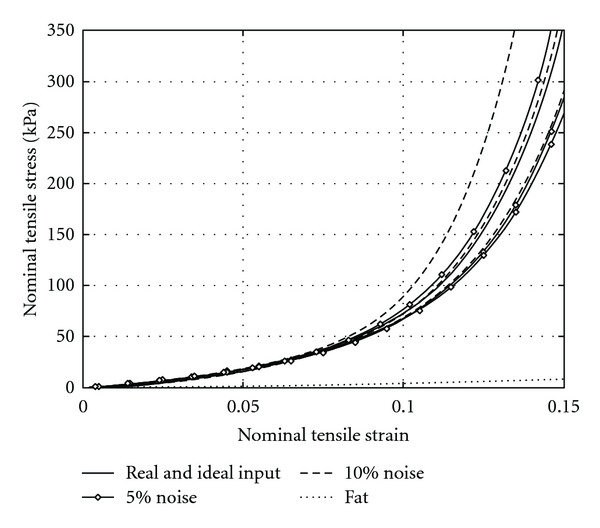
Nonlinear tensile strain-stress curves of the tumor as reconstructed from inputs with 5% and 10% noise.

The above elasto-mammography reconstructions are conducted using ideal inputs. However, noise is unavoidable in experimental data. To investigate the capability of the proposed nonlinear elasto-mammography modality and algorithm to handle imperfect experimental data, we conduct reconstruction using noisy input, where a randomly selected relative error between ±5% or ±10% is added to each displacement data in *U*_1_^*M*^. For each noise level, three case studies are conducted. The results are shown as noise 5% (I)–(III) and noise 10% (I)–(III) in Table 1, and the reconstructed tensile strain-stress curves of the tumor are plotted in Figure 7.

 It is observed that the strain-stress curves reconstructed with noisy input have similar shape to the ones with ideal input. It is not surprising that curves with 5% noise are closer to the real one than these curves with 10% noise. It demonstrated that, in order to get robust results, we need to make effort to decrease the noise in displacement measurements. It is noted that all the predicted strain-stress curves of tumor, with or without measurement noise, are well distinguished from the curve of fatty tissue (the lowest curve in Figure 7); that is, being much stiffer. That is, even though measurement noise exists, the tumor can be identified by recognizing the difference of stiffness between tumors and the surrounding tissues. This demonstrates that the nonlinear elasto-mammography results are accurate enough for diagnosis of tumors in clinical application.

The previous nonlinear elastography based on surface measurement [13] fails to reconstruct material parameters when ±5% random noise is added to the input. A regularization is required to provide additional constrain. In comparison, the present elasto-mammography yields accurate enough material parameters even with ±10% random noise. The reason is, as mentioned in Sections  4.1 and 4.2, that the displacements extracted on the surface of the tumor from mammography projections contain direct information of the mechanical properties of the tumor, which enhances the robustness of reconstruction and increases the accuracy, in particular of the tumor's parameters.

### 4.5. Advantages of Nonlinear Elasto-Mammography

In this study, a nonlinear elasto-mammography framework is developed to incorporate the conventional X-ray mammography for characterization of breast tissue properties. This work extends our previous study linear elasto-mammography [12] and nonlinear elastography [13]. Comparing with previous study, nonlinear elasto-mammography has the following three major advantages.

Imaging techniques: an imaging technique should be selected to measure deformation in elastography. In the proposed nonlinear elasto-mammography, the deformation is measured by conventional X-ray mammography while USE or MRI is applied in nonlinear elastography. Traditional X-ray has advantages of low cost and high resolution, compared with USE and MRI.

Deformation theory: the linear elasto-mammography framework is based on infinitesimal strain deformation theory. However, it is well known that the mechanical behavior of biological soft tissue is nonlinear. In nonlinear elasto-mammography, nonlinear material model and deformation theory are applied so that more accurate results could be obtained.

Inversion techniques: once displacements are measured, an inversion technique is applied to reconstruct elastic properties. In linear elasto-mammography, an adjoint method is applied and then a nonlinear adjoint method is developed for nonlinear elastography. In this study, the nonlinear adjoint method is further improved to enhance the numerical efficiency and stability of reconstruction of elastic properties.

Therefore, the proposed nonlinear elasto-mammography framework has advantage of imaging techniques, deformation theory, and inversion techniques. It combines the simplicity of projective X-ray mammography measurement with the accuracy of nonlinear elastography.

## 5. Summary

This study presents a nonlinear elasto-mammography method that combines elastography reconstruction and X-ray mammography imaging for the purpose of diagnosis of breast tumors by identification of the finite-strain mechanical parameters of breast tissues and tumors. The displacement information of selected material points is extracted from mammography projections before and after breast compression. Correspondingly, the previously developed nonlinear elastography algorithm has been adjusted with a revised adjoint gradient method to incorporate projection-type displacement measurement. The simulations with heterogeneous breast phantom proved the feasibility of elasto-mammography and tested the efficiency and robustness of the reconstruction algorithm. The simulations show that the deformation of the tumor, depicted by the projected displacement on the surface of the tumor extracted from mammography images, is critical for the success of elasto-mammography reconstruction.

## Figures and Tables

**Figure 8:**
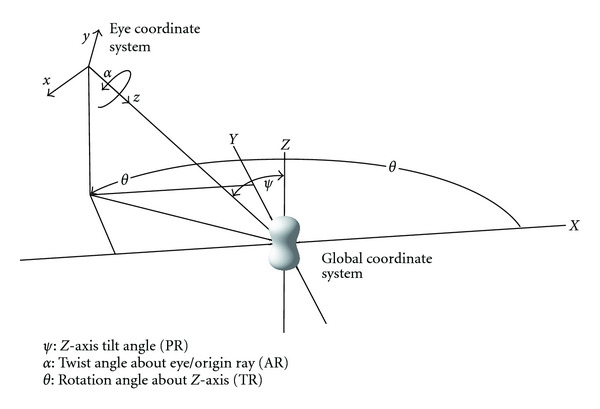
Illustration of global coordinate and eye coordinate. An object in global coordinates [*X*, *Y*, *Z*] is projected in eye coordinates [*x*, *y*, *z*]. The relation between direction vectors is dependent on *ψ*, *α*, and *θ*.

**Table 1:** Initial guess and nonlinear elasto-mammography reconstruction results of the fatty tissue, glandular tissue, and tumor in a 3D breast. The reconstructions are based on ideal mammography measurement, mammography measurement with ±5% and ±10% random noise, respectively. (*λ* and *μ* are dimensionless, *γ* is in kPa.)

	Fatty			Glandular			Tumor		
	*λ*_*f*_	*μ*_*f*_	*γ*_*f*_	*λ*_*g*_	*μ*_*g*_	*γ*_*g*_	*λ*_*d*_	*μ*_*d*_	*γ*_*d*_
Real	35	12.5	0.4	50	25	0.25	80	35	1.5
Guess	20	10	1	20	10	1	20	10	1
Ideal Input									
Reconstruction	35.00	12.50	0.40	50.00	25.00	0.25	79.83	34.93	1.51
5% Noise (I)									
Reconstruction	32.95	11.76	0.44	51.82	26.15	0.23	77.12	31.10	1.69
5% Noise (II)									
Reconstruction	34.82	12.35	0.41	51.62	26.10	0.23	66.14	29.57	1.88
5% Noise (III)									
Reconstruction	35.9	12.69	0.39	49.67	25.08	0.25	83.75	37.27	1.40
10% Noise (I)									
Reconstruction	35.14	12.68	0.40	48.87	24.40	0.26	107.59	35.56	1.41
10% Noise (II)									
Reconstruction	31.89	11.69	0.46	52.17	25.39	0.24	90.29	31.01	1.69
10% Noise (III)									
Reconstruction	36.75	12.89	0.37	48.30	24.54	0.26	107.20	48.89	0.92

## Appendices

### A. Displacement Transition between Coordinate Systems

This appendix presents the transition of a displacement vector between global coordinates and projection coordinates. The outcome is the projection matrix **R** in formulas (5) and (7).

To be consistent with computational geometry, we call the projection coordinates as *eye coordinates*. As illustrated in Figure 8, the global coordinates are denoted as [*X*, *Y*, *Z*] and eye coordinates are [*x*, *y*, *z*]. Their direction vectors are [**e**_*X*_, **e**_*Y*_, **e**_*Z*_]^*T*^ and [**e**_*x*_′, **e**_*y*_′, **e**_*z*_′]^*T*^, respectively. As shown in Figure 8, the eye coordinates rotate from global coordinates by three angles: *Z*-axis tilt angle *ψ*, twist angle about eye/original ray *α*, and rotation angle about *Z*-axis *θ*. It can be shown that

(A1)
$$\left(\begin{array}{c} e_{x}^{\prime }\\ e_{y}^{\prime }\\ e_{z}^{\prime } \end{array}\right)=\left[Q\right]\left(\begin{array}{c} e_{x}\\ e_{y}\\ e_{z} \end{array}\right),$$

where the rotation matrix *Q* is

(A2)
$$\left[Q\right]=\left[\begin{array}{ccc} \cos\alpha \cdot \cos\theta \cdot \cos\psi -\sin\alpha \cdot \sin\theta & \cos\theta \cdot \sin\alpha +\cos\alpha \cdot \cos\psi \cdot \sin\theta & \cos\alpha \cdot \sin\psi \\ -\cos\theta \cdot \cos\psi \cdot \sin\alpha -\cos\alpha \cdot \sin\theta & \cos\alpha \cdot \cos\theta -\cos\psi \cdot \sin\alpha \cdot \sin\theta & -\sin\alpha \cdot \sin\psi \\ \cos\theta \cdot \sin\psi & \sin\theta \cdot \sin\psi & -\cos\psi \end{array}\right].$$

Now, consider a displacement vector **u** of a material point from undeformed position to deformed position. The global coordinates of **u** are {*u*_*X*_, *v*_*Y*_, *w*_*Z*_}, the eye coordinates are {*u*_*x*_, *v*_*y*_, *w*_*z*_}, and their relationship can be derived as

(A3)
$$\left(\begin{array}{c} u_{x}\\ v_{y}\\ w_{z} \end{array}\right)=\left[Q\right]\left(\begin{array}{c} u_{X}\\ v_{Y}\\ w_{Z} \end{array}\right).$$

In mammography projection, the displacement component in **e**_*z*_′ direction, *w*_*z*_, is not obtainable, and only *u*_*x*_ and *v*_*y*_ are measured. Therefore, (A3) reduces to

(A4)
$$\left(\begin{array}{c} u_{x}\\ v_{y} \end{array}\right)=\underset{\left[Q^{\prime }\right]}{\underset{︸}{\left[\begin{array}{ccc} \cos\alpha \cdot \cos\theta \cdot \cos\psi -\sin\alpha \cdot \sin\theta & \cos\theta \cdot \sin\alpha +\cos\alpha \cdot \cos\psi \cdot \sin\theta & \cos\alpha \cdot \sin\psi \\ -\cos\theta \cdot \cos\psi \cdot \sin\alpha -\cos\alpha \cdot \sin\theta & \cos\alpha \cdot \cos\theta -\cos\psi \cdot \sin\alpha \cdot \sin\theta & -\sin\alpha \cdot \sin\psi \end{array}\right]}}\left(\begin{array}{c} u_{X}\\ v_{Y}\\ w_{Z} \end{array}\right)=\left[Q\prime \right]\left(\begin{array}{c} u_{X}\\ v_{Y}\\ w_{Z} \end{array}\right).$$

Finally, the FE solution of displacement field *u*_1_, when projected, becomes **R***u*_1_ where **R** is the assemble of [*Q*′] according to the FE discretization and assembling methods.

### B. Adjoint Method for Gradients of Objective Function

Direct calculation of the gradients ∂Φ/∂**p** of the objective function involved in the minimization-based parametric identification is difficult, because *u*_1_ is an implicated function of **p**. An adjoint method will be derived here for efficient and analytical calculation of the gradients. To release the implicit coupling between *u*_1_ and **p**, we introduce the constraint (1) into the objective function (5) and obtain a Lagrangian:

(B1)
$$\begin{array}{c} L={\left(Ru_{1}-U_{1}^{M}\right)}^{T}\Lambda \left(Ru_{1}-U_{1}^{M}\right)+{\left\{\begin{array}{c} w_{1}\\ w_{2} \end{array}\right\}}^{T}\left\{\begin{array}{c} f_{1}^{\text{in}}-f_{1}^{\text{out}}\\ f_{2}^{\text{in}}-f_{2}^{\text{out}} \end{array}\right\}, \end{array}$$

where *w*_1_ and *w*_2_ are arbitrary virtual displacements. In this Lagrangian, *u*_1_ and **p** are explicit variables and are no longer coupled. It is noted that Φ = *L* and *δ*Φ = *δL* for arbitrary *w*_1_ and *w*_2_ under the constraint (1). The variation *δL* can be expressed as

(B2)
$$\begin{array}{cc} \delta L & =2{\left(Ru_{1}-U_{1}^{M}\right)}^{T}\Lambda \left(R\delta u_{1}\right)\\ & \;+\left(w_{1}^{T}K_{11}^{\text{in}}-w_{1}^{T}K_{11}^{\text{out}}+w_{2}^{T}\frac{\partial f_{2}^{\text{in}}}{\partial u_{1}}-w_{2}^{T}\frac{\partial f_{2}^{\text{out}}}{\partial u_{1}}\right)\delta u_{1}\\ & \;+w_{1}^{T}\frac{\partial f_{1}^{\text{in}}}{\partial p}\delta p+w_{2}^{T}\frac{\partial f_{2}^{\text{in}}}{\partial p}\delta p-w_{2}^{T}\frac{\partial f_{2}^{\text{out}}}{\partial p}\delta p \end{array}$$

for which the equality constraint (1) has been applied. Note that the prescribed external force *f*_1_^out^ is independent of **p**. Equation (B2) can be further simplified by letting the arbitrary virtual displacement *w*_2_ = 0, as

(B3)
$$\begin{array}{cc} \delta L & =\left\{2{\left(Ru_{1}-U_{1}^{M}\right)}^{T}\Lambda R+w_{1}^{T}K_{11}^{\text{in}}-w_{1}^{T}K_{11}^{\text{out}}\right\}\delta u_{1}\\ & \;+w_{1}^{T}\frac{\partial f_{1}^{\text{in}}}{\partial p}\delta p. \end{array}$$

If we select a *w*_1_ to let {2(**R***u*_1_ − *U*_1_^*M*^)^*T*^Λ**R** + *w*_1_^*T*^*K*_11_^in^ − *w*_1_^*T*^*K*_11_^out^}*δu*_1_ = 0 for arbitrary *δu*_1_, we obtain a simplest form of *δL*, as

(B4)
$$\delta L=w_{1}^{T}\frac{\partial f_{1}^{\text{in}}}{\partial p}\delta p=\left(w_{1}^{T}\frac{\partial f_{1}^{\text{in}}}{\partial p}+w_{2}^{T}\frac{\partial f_{2}^{\text{in}}}{\partial p}\right)\delta p\;\left(w_{2}=0\right).$$

Consider that *δ*Φ = *δL* for arbitrary *w*_1_ and *w*_2_, we obtain (6) in the text with the following selection of *w*_1_ and *w*_2_:

(B5)
$$\begin{array}{c} \left(K_{11}^{\text{in}}-K_{11}^{\text{out}}\right)w_{1}=K_{11}^{\text{eff}}w_{1}=-2R^{T}\Lambda \left(Ru_{1}-U_{1}^{M}\right),\\ w_{2}=0 \end{array}$$

which is (7) in the text.

By introducing the adjoint method, it seems that more equations (B5) and variables (*w*_1_ and *w*_2_) are involved. But the solution of (B5) is straightforward and the computational cost is minimal, because *K*_11_^eff^ has been computed and factorized when solving for the displacement *u*_1_ as in (3).

The gradients ∂Φ/∂**p** can also be calculated directly as

(B6)
$$\frac{\partial \Phi }{\partial p}=2{\left(Ru_{1}-U_{1}^{M}\right)}^{T}\Lambda R\frac{\partial u_{1}}{\partial p},$$

in which ∂*u*_1_/∂**p** can be computed numerically using finite-different method:

(B7)
$$\frac{\partial u_{1}}{\partial p}\approx \frac{u_{1}\left(p+\delta p\right)-u_{1}\left(p\right)}{\delta p}\;\left(\delta p\;\text{is}\;\text{a}\;\text{small}\;\text{change}\;\text{of}\;p\right)$$

or analytically by solving linear equations:

(B8)
$$K_{11}^{\text{eff}}\frac{\partial u_{1}}{\partial p}=-\frac{\partial f_{1}^{\text{in}}}{\partial p}.$$

For finite-strain nonlinear problem, the finite-different method is unaffordable due to the high computational expense to solve (1) for *u*_1_. Solving (B8) is straightforward and is much less expensive for *K*_11_^eff^ has been computed and factorized. However, (B8) needs to be solved for every material parameters involved; for example, in the exemplar simulations in this work, it needs to be solved nine times because each material has three parameters. In comparison, the proposed adjoint method (B5), (B6) requires only one solution for *w*_1_, regardless of the number of material parameters involved.
